# Simulated herbivory enhances leaky sex expression in the dioecious herb *Mercurialis annua*

**DOI:** 10.1093/aob/mcab129

**Published:** 2021-10-20

**Authors:** Nora Villamil, Xinji Li, Emily Seddon, John R Pannell

**Affiliations:** 1 Department of Ecology and Evolution, Université de Lausanne, Switzerland; 2 Vegetation Ecologist, NatureServe, Boulder, CO 80301, USA

**Keywords:** Herbivory, sexual system, reproduction, leakiness, sex inconstancy, hermaphroditism, wind pollination, self-fertilization, mating system, size-dependent sex allocation, monoecy, dioecy

## Abstract

**Background and Aims:**

Plant reproductive traits are widely understood to be responsive to the selective pressures exerted by pollinators, but there is also increasing evidence for an important role for antagonists such as herbivores in shaping these traits. Many dioecious species show leaky sex expression, with males and females occasionally producing flowers of the opposite sex. Here, we asked to what extent leakiness in sex expression in *Mercurialis annua* (Euphorbiaceae) might also be plastically responsive to simulated herbivory. This is important because enhanced leakiness in dioecious populations could lead to a shift in both the mating system and in the conditions for transitions between combined and separate sexes.

**Methods:**

We examined the effect of simulated herbivory on the sexual expression of males and females of *M. annua* in two experiments in which different levels of simulated herbivory led to enhanced leakiness in both sexes.

**Key Results:**

We showed that leaky sex expression in both males and females of the wind-pollinated dioecious herb *M. annua* is enhanced in response to simulated herbivory, increasing the probability for and the degree of leakiness in both sexes. We also found that leakiness was greater in larger females but not in larger males.

**Conclusions:**

We discuss hypotheses for a possible functional link between herbivory and leaky sex expression, and consider what simulated herbivory-induced leakiness might imply for the evolutionary ecology of plant reproductive systems, especially the breakdown of dioecy and the evolution of hermaphroditism.

## INTRODUCTION

Floral morphology and other reproductive traits are often strongly influenced by selection due to interactions with pollinators (Willmer, 2011). However, reproductive traits can also be shaped by selection due to antagonists such as herbivores (Ashman, 2002; Steets and Ashman, 2004; Carr and Eubanks, 2014; Johnson *et al.*, 2015). For instance, the strength of selection due to herbivores was found to be equal to or stronger than that due to pollinators in 67 % of species in which the role of both mutualists and antagonists had been studied (Johnson *et al.*, 2015). There is also growing evidence for the co-ordinated evolution of plant defensive and reproductive traits (Strauss and Whittall, 2006; Rausher, 2008; Ågren *et al.*, 2013; Campbell and Kessler, 2013; Carr and Eubanks, 2014; Campbell, 2015; Johnson *et al.*, 2015). Specifically, herbivores, and plant responses to them, have been found to affect the floral display (Strauss *et al*., 1996, 1999; Herrera, 2000; Parachnowitsch and Caruso, 2008; Ågren *et al.*, 2013), flower colour (Strauss *et al.*, 2004; Vaidya *et al.*, 2018), flowering time (Parachnowitsch and Caruso, 2008), floral morphology (Galen, 1999; Galen and Cuba, 2001; Santangelo *et al.*, 2019), floral scents and pollinator rewards (Kessler and Halitschke, 2009; Kessler *et al.*, 2011; Ramos and Schiestl, 2019; Aguirre *et al.*, 2020), mating systems (Steets and Ashman, 2004; Ivey and Carr, 2005; Penet *et al.*, 2009; Kessler *et al.*, 2011) and sex allocation (reviewed by Ashman, 2002). Yet despite the increasing evidence for the importance of herbivory in the ecology and evolution of plant reproduction, very little attention has been given to the effect of herbivory on sex allocation and other traits that might be important in evolutionary transitions between hermaphroditism and dioecy.

Herbivores can affect sex allocation patterns either directly via plant size or indirectly via effects on pollinator behaviours. By reducing plant size and resource availability, herbivory in dioecious populations may directly increase mortality of individuals with the more costly sex function, potentially leading to or enhancing sex ratio biases (Cornelissen and Stiling, 2005; Sánchez-Vilas and Pannell, 2011*b*; Geber *et al.*, 2012). For similar reasons, herbivory in hermaphroditic species can cause individuals to shift their investment towards the least costly sexual function (Diggle, 1994; Seger and Eckhart, 1996; Ashman, 2002; Zhang and Jiang, 2002; West, 2009; Hirata *et al.*, 2019). A particularly interesting and important possibility is that herbivory could influence the sex allocation of males and females of dioecious species by altering their tendency to produce flowers of the opposite sex through ‘leaky’ or ‘inconstant’ sex expression.

Leaky sex expression has been described in at least 40 dioecious species, including gymnosperms and angiosperms (Ehlers and Bataillon, 2007; Cossard and Pannell, 2019), but it is probably more frequent. In some cases, it may simply reflect the rudimentary expression of hermaphroditism in species that have not completed the transition to fully separate sexes (Delph, 2003; Delph and Wolf, 2005). However, it may also be maintained by selection for reproductive assurance under conditions in which mating opportunities are limited (Crossman and Charlesworth, 2014; Cossard and Pannell, 2021; Cossard *et al.*, 2021). Mate limitation could arise from colonizing new, mate-less environments (Baker, 1965; Pannell, 2015) or from pollinator limitation caused by biotic (e.g. herbivory) or abiotic factors (e.g. environmental heterogeneity, such as across altitudinal gradients) (Kessler *et al.*, 2011; Trøjelsgaard and Olesen, 2013). Until recently, almost nothing was known about the developmental basis of leakiness, but several studies indicate that it may have a plastic component, with plants responding to biotic or abiotic factors (Freeman *et al.*, 1980; Bierzychudek and Eckhart, 1988; Sakai *et al.*, 1995). For example, in some species, cool and humid conditions at high altitudes have been found to favour males and strict dioecy (unisexuality), whereas warmer, drier conditions at lower altitudes favour hermaphrodites and enhanced leakiness, showing a leakiness response to environmental cues (Delph, 1990*b*; Sakai and Weller, 1991; Humeau *et al.*, 1999; Venkatasamy *et al.*, 2007). A recent study of leaky sex expression in the wind-pollinated dioecious plant *Mercurialis annua* found that leakiness can also respond plastically to plant–plant interactions and population sex ratios (Cossard and Pannell, 2021), such that females deprived of pollen-producing mates are more likely to produce male flowers than comparable females receiving abundant pollen (Cossard and Pannell, 2021).

Here, we asked to what extent leakiness in sex expression in *M. annua* might also be plastically responsive to wounding through simulated herbivory and associated tissue loss. This is important because enhanced leakiness in dioecious populations could lead to a shift in both the mating system and the conditions for transitions between combined and separate sexes. Our study also addresses a perceived gap in our understanding, identified by Johnson *et al.* (2015), of how herbivore-induced changes in phenotype might alter a species’ mating system. Most documented cases of the effect of herbivory on the mating system point to reduced selfing in hermaphrodite species (reviewed by Johnson *et al.*, 2015), whereas enhanced leakiness in dioecious species would potentially allow selfing instead. Previously, Yampolsky (1930) and Kuhn (1939) found that pruning individuals of *M. annua* tended to enhance the production of flowers of the opposite sex, but neither study characterized this response in any detail.

Wounding and tissue loss due to herbivory might affect leakiness in sex expression for a number of reasons. First, a plant’s response to herbivory might include altering its endogenous hormone balance (Thaler *et al.*, 2001; Ballaré, 2011; Robert-Seilaniantz *et al.*, 2011; Naseem *et al.*, 2015), with potential pleiotropic effects on its sex expression (Riemann *et al.*, 2003; Wasternack *et al.*, 2013; Yuan and Zhang, 2015). Second, wounding and tissue loss might affect a plant’s sex expression via effects on plant size or resource status, as predicted by theories of size- or resource-dependent sex allocation (Ghiselin, 1969; Trivers and Willard, 1973; Charnov, 1979, 1982; Freeman *et al.*, 1980; Warner, 1988; de Jong and Klinkhamer, 1989; Klinkhamer *et al.*, 1997; reviewed by West, 2009). For instance, if physical injuries reduce an individual’s resource status, selection might favour a strategy that includes a shift in sex allocation towards the cheaper sex function. While studies of size- and/or resource-dependent sex allocation and gender (Lloyd, 1980) have tended to focus on hermaphrodites (Freeman *et al.*, 1980; Korpelainen, 1998; Vega-Frutis *et al.*, 2014), the same ideas might apply to sex inconstancy in dioecious species.

We examined the effect of simulated herbivory on the sexual expression of males and females of *M. annua* in two experiments in which different levels of simulated herbivory led to enhanced leakiness in both sexes. In females, we compared leakiness levels between control (undamaged) and damaged females. In males, we quantified leakiness of plants under a low and a high simulated herbivory treatment. We used the data from both experiments to address the following questions. (1) How does simulated herbivory affect the probability of leakiness in males and females of a dioecious species? (2) How does simulated herbivory affect the number of opposite sex reproductive structures produced by males and females of a dioecious species? (3) To what extent are the male and female changes in sex expression in response to simulated herbivory mediated by plant size?

In both of our experiments, our simulated herbivory treatments involved the removal of a given proportion of the primary shoot through cutting, i.e. it involved both wounding and associated tissue loss. This treatment differs from natural herbivory in several important respects. For instance, experimental cutting of the shoot is not associated with the potential chemical and/or microbial stimuli present in the herbivores’ saliva or mouth, to which plants are known to have evolved specific defence responses. Damage inflicted by a simple quick cut also differs from the protracted accumulative damage inflicted by certain invertebrates that may spend long periods on a given plant. Nevertheless, to a certain extent, our cutting treatment resembles the herbivory suffered by *M. annua* plants under field conditions: they are often damaged by slugs that prune off a section of the main axis before consuming all or part of it (pers. obs.). Previous experiments with *M. annua* subjected males and females to herbivory by snails and revealed sexually dimorphic responses (Sánchez-Vilas and Pannell, 2011*b*). Such experiments are valuable, but natural herbivory treatments are also associated with potential effects of increased leaf temperature due to insect caging, and they also sacrifice the advantages of experimental control over the timing, intensity and uniformity of damage caused (Hjältén, 2008). Our experiment benefited from these elements of control, not least because they allowed a relatively uniform reduction of plant size and corresponding resource status. Nevertheless, it is important to recognize that our ‘simulated herbivory’ treatment is probably a poor imitation of natural herbivory, even though mechanical wounding and tissue removal have been validated as a suitable approach to study biotic interactions at small scales, such as the consequences of herbivores on plant growth, defence, physiology and resource allocation (Hjältén, 2008).

## MATERIALS AND METHODS

### Study system

*Mercurialis annua* is a polyploid complex of wind-pollinated ruderal herbs that occupy disturbed habitats across eastern, central and western Europe (Tutin *et al.*, 1968; Obbard *et al.*, 2006). Diploid populations are dioecious, with an XY chromosomal system of sex determination (Russell and Pannell, 2015; Veltsos *et al*., 2018, 2019; Li *et al.*, 2019). Males produce staminate flowers on pedunculate inflorescences held above the plant. Females produce two- to three-ovulate flowers on sub-sessile pedicels in the leaf axils (Tutin *et al.*, 1968). In addition to these differences in the floral sex and inflorescence morphology between males and females, the sexes also differ in a number of vegetative characters, including plant and root biomass, patterns of resource allocation to growth and reproduction throughout their development and their competitive abilities (Sánchez Vilas and Pannell, 2011*a*; Sánchez-Vilas *et al.*, 2011; Tonnabel *et al.*, 2017). As in the case of many dioecious plants (Ehlers and Bataillon, 2007; Cossard and Pannell, 2019), dioecious *M. annua* shows leakiness in sex expression, with both males and females occasionally producing fully functional flowers of the opposite sexual function (Pannell *et al.*, 2008; Cossard and Pannell, 2019, 2021).

In the field, *M. annua* plants are attacked mainly by generalist herbivores. Within their native distribution range, between Morocco and the Iberian Peninsula, plants are subject to moderate levels of herbivory, mainly by snails of the genus *Cepaea*; outside their native range, herbivores also include *Helix aspersa* snails (Sánchez-Vilas and Pannell, 2011*b*). As is common in species with separate sexes (Ågren *et al.*, 1999), *M. annua* shows male-biased herbivory, and plants display sexual dimorphism in response to wounding and tissue loss (Sánchez-Vilas and Pannell, 2011*b*) and other stressors (Sánchez Vilas and Pannell, 2011*a*; Sánchez-Vilas *et al.*, 2011; Orlofsky *et al.*, 2016).

### Plant culture

Plants of the diploid *M. annua* were sown and grown within a polytunnel under controlled conditions at the University of Lausanne, Switzerland. The experiment with female plants was established during March 2016, while the experiment with male plants was established in May of the same year. In both experiments, plants were sown and raised to maturity in seedling trays. When plants reached reproductive maturity, plants of the desired sex for the respective experiment were repotted in pots with soil (Ricoter substrate 140) and slow-release fertilizer (Hauert Tardit 6M pellets; 5 g fertilizer L^–1^ of soil). Plants were subjected to the simulated herbivory treatments and allowed to regrow for 10 weeks in the male experiment and for 8 weeks in the female experiment. After this period, plants were harvested, and the numbers of male and female flowers were recorded. Plants were then dried and weighed.

### Simulated herbivory treatments

For the male experiment, individuals were grown in pairs in pots to save space (with pot thus being the unit of replication; *n* = 828 pots). Both individuals in a given pot were subjected to the same low or high herbivory treatment. Over a period of 10 weeks of growth, plants under the low-herbivory treatment were pruned once, removing all tissue above the first internode (3 cm above the soil surface), whereas plants under the high herbivory treatment were pruned twice within these 10 weeks, on both occasions by removing all tissue above the first internode. For the female experiment, individuals were transplanted into individual pots (*n* = 219 plants). For plants under the herbivory treatment, the apical section (10 cm) of the main stem was pruned once, removing all plant tissue above the cut point; plants under the control treatment were left intact.

Our experiment on male plants involved a comparison between two treatments of simulated herbivory of contrasting intensity, but did not include undamaged plants. This is because it was initially part of a study that specifically aimed at generating males with leaky sex expression for the production of YY male plants (Li *et al.*, 2019). The absence of undamaged males means that our low vs. high herbivory comparison is a more conservative estimate of sensitivity to the intensity of herbivory, as confirmed by previously documented levels of leaky sex expression for undamaged males of the same population (Cossard and Pannell, 2019; see the Discussion for details). The male experiment thus specifically asks how sensitive plants are in their leakiness to the degree of simulated herbivory rather than to herbivory as a categorical variable.

### Statistical analyses

Statistical analyses were conducted in R version 3.6.1 (R Core Team, 2016). Models were fitted using ‘lme4’ R package (Bates *et al.*, 2015), unless otherwise stated, with residuals evaluated with the ‘DHARMa’ R package (Hartig and Hartig, 2017).

To test whether the simulated herbivory treatment affected the probability of sex change in males and females, we fitted generalized linear binomial models. For males, the presence or absence of seeds was considered as the response variable, with the herbivory treatment and biomass fitted as predictors. For females, the presence or absence of male flowers was the response variable, and the predictors were the simulated herbivory treatment, plant biomass and plant population.

To test the effect of simulated herbivory on the number of reproductive structures of the opposite sex, we fitted two separate Poisson models. We tested and accounted for zero inflation in both models. Seed production in males was zero inflated and was consequently analysed with a Poisson zero-inflated mixed model using the ‘glmmADMB’ package (Skaug *et al.*, 2011). The number of seeds produced by males was fitted as the response variable, with the herbivory treatment and plant biomass fitted as fixed effects. To control for overdispersion, we also included an observation-level random effect (OLRE) (Hinde, 1982; Harrison, 2014). The model testing the effect of simulated herbivory on male flower production by females was not zero inflated. Here, we fitted the number of male flowers produced by females as the response variableand the herbivory treatment and plant biomass as fixed effects, and included an OLRE.

## RESULTS AND DISCUSSION

### *Patterns of leakiness in sex expression in* M. annua

**Fig. 1.**
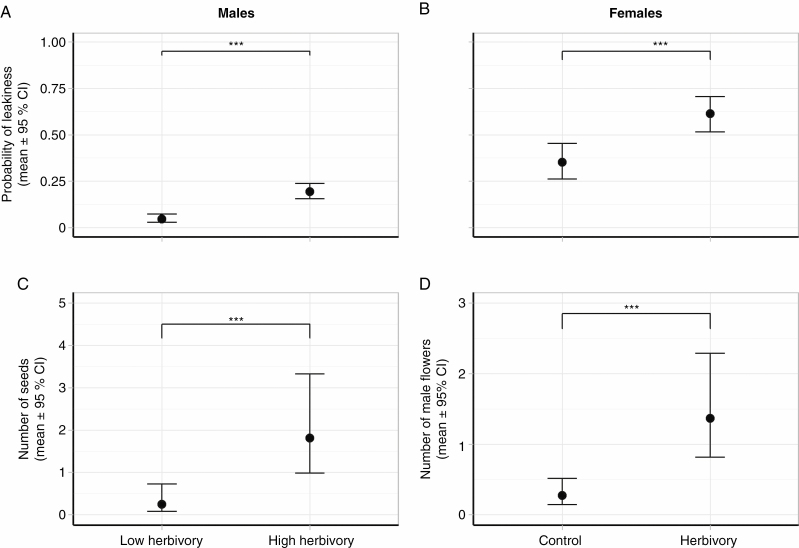
Leakiness in sex expression in response to simulated herbivory for males and females of *Mercurialis annua*, in terms of effects on the probability of leakiness (A and B), and in the number of reproductive structures of the opposite sex (C and D). Error bars represent 95 % confidence intervals, and asterisks indicate significant differences (*P* < 0.0001).

Simulated herbivory significantly increased the probability and the degree of leakiness in both males and females (Table 1; Fig. 1; Supplementary data Table S1). Thus, males in pots under high herbivory were 15 % more likely to produce seeds than those under low herbivory (Fig. 1A) and they produced 13 times more seeds on average (Fig. 1C; Table 1). Similarly, females under simulated herbivory were 26 % more likely to produce male flowers than control females (Fig. 1B), and they produced five times more male flowers than control females (Fig. 1D; Table 1). We also found that while females were 0.51 % more likely to produce male flowers and produced 1.06 more male flowers per gram of additional biomass, male leakiness did not depend on plant size (Table 1).

**Table 1.** Model output for the effects of simulated herbivory on leakiness in sex expression in males and females of *Mercurialis annua*

Response variable	Sex	Fixed effects	n	LRT	*P*-value		Random effects	Variance	Error distribution
Probability of leakiness	Males	Herbivory	828	35.49	2.55^−09^	***	NA	NA	Binomial
		Biomass		0.002	0.96	n.s.			
Probability of leakiness	Females	Herbivory		29.34	4.25^−07^	***	NA	NA	Binomial
		Biomass	219	23.00	1.61^−06^	***			
		Population		0.93	0.81	n.s.			
Number of seeds	Males	Herbivory	828	43.06	5.29^−11^	***	OLRE	0.64	Poisson zero inflated
		Biomass		0.94	0.32	ns			
Number of male flowers	Females	Herbivory		19.01	1.29^−05^	***	OLRE	4.96	Poisson
		Biomass	219	32.32	1.30^−08^	***			
		Population		5.16	0.16	n.s.			

LRT, likelihood ratio test; OLRE, observation-level random effect, added to control for overdispersion in Poisson models. Text in bold indicates significant terms *P* < 0.05.

While leakiness in sex expression can affect both sexes of dioecious species, generally males are more likely to be leaky than females, a pattern that may reflect incomplete transitions from hermaphroditism to dioecy via gynodioecy, with males retaining a residual female function (Ehlers and Bataillon, 2007; Cossard and Pannell, 2019). Against this background, the greater probability of leakiness in females of *M. annua* is unusual. Our results confirm greater leakiness in females than in males in *M. annua*, and they also indicate that under simulated herbivory this pattern of enhanced leakiness is maintained and even accentuated. Previous findings on the baseline percentage probability of sex inconstancy in undamaged *M. annua* males of the same source population were 3 % (Cossard and Pannell, 2019), compared with 4.5 % in males under the low herbivory and 23.8 % under the high herbivory treatment.

Dioecy is ancestral and well established across the genus *Mercurialis*, and there is no indication that separate sexes evolved via gynodioecy (though they might have done so). Greater leakiness in females than males might reflect selection for reproductive assurance, which would favour the maintenance of leaky sex expression in females rather than males, because a small amount of pollen produced by females might suffice for the production of a large number of seeds, in contrast to the few seeds produced by males (Pannell and Barrett, 2001; Cossard and Pannell, 2019). This might also explain why we found that males, when leaky, tend to express a greater degree of leakiness than females, as found previously by Cossard and Pannell (2019). These patterns of leakiness in *M. annua* are thus consistent both with the expectations of range expansion or metapopulation models, in which new populations are frequently established by single individuals, and with the ruderal habit and metapopulation structure of *M. annua* across Europe (Pannell and Barrett, 2001; Obbard *et al.*, 2006; Eppley and Pannell, 2007).

Our results shed further light on the expression of leakiness in sex expression in *M. annua*, to our knowledge the only plant to date whose leakiness has been investigated experimentally in quantitative terms, showing that antagonists can affect this reproductive trait. Leaky sex expression is common in dioecious species, but the basis of variation in leakiness among individuals has remained almost entirely obscure. Our current and previous studies of leaky sex expression in *M. annua* indicate that the phenomenon cannot be attributed only to developmental instability or poorly canalized separation of the sexes. Rather, leakiness is clearly a more complex trait, with components of variation attributable not only to genetic differences among individuals (Cossard *et al.*, 2021) but also to phenotypic plasticity. Previous work found that females of *M. annua* were more likely to express a male function when growing under conditions of pollen (or mate) limitation (Cossard and Pannell, 2021). By showing that leaky sex expression in *M. annua* also responds to simulated herbivory, our current study now adds further evidence for the contribution of phenotypic plasticity to phenotypic variation in sex expression and highlights the role that antagonists can have in shaping reproductive traits in angiosperms. Our findings also provide evidence reinforcing the idea of co-ordinated evolution between defensive and reproductive traits in angiosperms showing herbivory-induced leakiness in male and female plants.

Even though simulated herbivory or plant wounding differs from natural herbivory in several aspects, controlled, simulated herbivory has been a significant research tool to understand plant responses to damage, mainly by disentangling stimuli types and intensities (Hjältén, 2008; Lehtilä and Boalt, 2008; Waterman *et al.*, 2019). A meta-analysis comparing the effects of natural and simulated herbivory on plant responses reported no significant difference in a considerable proportion of studies and statistical tests, leading the authors to endorse simulated herbivory as a valid methodological approach (Lehtilä and Boalt, 2008). Yet they found that not all plant responses were equally sensitive to the type of herbivory. Phytochemicals and defensive compounds were the most sensitive responses to the type of herbivory treatment (85 % of studies showed significant differences), whilst plant growth and reproduction were among the least sensitive plant responses to natural and simulated herbivory (20–30 % of the studies/tests showed significant differences) (Lehtilä and Boalt, 2008). This meta-analysis also showed that, in most cases, natural herbivory has stronger effects than simulated herbivory, so that effects observed through simulated herbivory probably represent underestimates of what could be expected in the wild (Lehtilä and Boalt, 2008). Our study evaluated the effects of simulated herbivory on plant sexual expression, a plant-level response linked to growth and reproduction. Although it would seem from the analysis of Lehtilä and Boalt (2008) that our experimental approach was likely to be suitable to address our questions, we expect that natural herbivory would have elicited somewhat different (and perhaps larger) responses. Further research with more natural herbivory treatments would be worthwhile.

### Why should leakiness in sex expression be sensitive to simulated herbivory?

While it seems plausible that the sensitivity of leaky sex expression of unisexual plants to mate limitation might have evolved in response to selection for reproductive assurance in *M. annua* (Cossard and Pannell, 2021), a functional explanation for the sensitivity of leakiness to simulated herbivory is less obvious. Could it be that simulated herbivory-enhanced leakiness functions as a reproductive assurance mechanism too, for example if herbivory compromised mate availability? In most dioecious species, herbivory is male biased (Ågren *et al.*, 1999; Cornelissen and Stiling, 2005; Geber *et al.*, 2012), including in *M. annua* (Sánchez-Vilas and Pannell, 2011*b*), so that at least females might gain from induced leakiness in heavily damaged populations. Hesse and Pannell (2011) found that isolated females of *M. annua* were indeed pollen limited in the field, but we have no evidence that herbivory frequently brings about such isolation in *M. annua*, nor indeed how effectively the observed levels of leakiness in our experiment would actually restore seed production to pollen-limited females. Another possibility is that enhanced leakiness in response to simulated herbivory might be a collateral effect of hormonal changes resulting from the activation of plant defensive pathways. Plant hormones such as jasmonates are known to regulate both sex determination and defensive responses in a number of plants (Robert-Seilaniantz *et al.*, 2011; Yuan and Zhang, 2015), and it thus seems plausible that damage-induced hormonal changes might have altered the balance of sex-determining hormones in our experiment with *M. annua.* Although *M. annua* has chromosomal sex determination (Russell and Pannell, 2015; Veltsos *et al.*, 2018), its sex expression appears to be mediated by the endogenous levels of cytokinin and auxin (Hamdi *et al.*, 1987; Louis *et al.*, 1990; Durand and Durand, 1991; Li *et al.*, 2019) as well as by its exogenous application (Hamdi *et al.*, 1987; Durand and Durand, 1991).

Larger females, but not larger males, were more likely to produce flowers of the opposite sex, or to produce more of them (Table 1). There would seem to be two potential implications for this differential relationship between plant biomass and leakiness in males and females. First, our result reinforces the idea that in *M. annua* male flower production is costlier than female flower and fruit production, possibly because of the high investment of nitrogen in pollen (Harris and Pannell, 2008; Van Drunen and Dorken, 2012; Wright and Dorken, 2014). In this context, the fact that we found lower levels of leakiness in smaller individuals only for females is consistent with maleness being the costlier sex in *M. annua*, and with the expectation that larger individuals should allocate more towards the costlier sex because of their greater budget (Delph, 1990*a*; Seger and Eckhart, 1996; Klinkhamer *et al.*, 1997; Zhang and Jiang, 2002; West, 2009). Second, because larger plants haver higher siring success in *M. annua* (Tonnabel *et al.*, 2019), females might benefit from investing in pollen production only when large. In wind-pollinated herbs more generally, large size (in terms of height, which is correlated with biomass) may also directly benefit male function more than female function by promoting the dispersal of pollen from above the plant canopy (Klinkhamer *et al.*, 1997; Friedman and Barrett, 2009; Harder and Prusinkiewicz, 2013; Tonnabel *et al.*, 2019).

### Implications for plant mating and sexual system evolution

Although we remain largely ignorant of how selection might have shaped the interaction between leakiness in sex expression in *M. annua* and its responses to simulated herbivory, there are nevertheless several implications of this interaction for the species’ mating system and potential transitions between sexual systems, which have been frequent in annual lineages of the genus *Mercurialis* (Pannell *et al.*, 2008; Pannell, 2018). Most immediately, the induction of higher levels of leakiness by simulated herbivory in our experiment suggests that plant damage in natural populations may allow some degree of selfing (and thus mixed mating) in a species that would otherwise be fully outcrossing, as dioecious species are.

Our results contribute to a growing picture of the co-ordinated or interacting nature of mating system and defence evolution in plants (Carr and Eubanks, 2014; Johnson *et al.*, 2015; Lucas-Barbosa, 2016). For instance, Johnson *et al.* (2015) suggested that herbivory could shape the evolution of selfing from outcrossing as a result of herbivore-mediated inbreeding depression, and by affecting pollinator visitation via changes to flowers, potentially leading to pollen limitation. Most previous work has focused on species with combined sexes, and we are unaware of studies on how herbivory might affect reproduction in dioecious species, beyond the observation of (typically) male-biased susceptibility to damage (Ågren *et al.*, 1999; Cornelissen and Stiling, 2005; Geber *et al.*, 2012). If herbivory commonly induces leakiness and facultative selfing, as seems to be the case in *M. annua*, then the implications of herbivory for the mating system in dioecious species might differ from that in hermaphroditic species, in which herbivores have been found more typically to promote greater outcrossing (Johnson *et al.*, 2015).

Transitions from hermaphroditism to dioecy were long seen as evolutionary dead ends (Heilbuth, 2000; Heilbuth *et al.*, 2001; Käfer *et al*., 2014, 2017), but a recent comparative analysis suggests that reversions to hermaphroditism may have been common (Muyle *et al.*, 2020). In *Mercurialis* in particular, monoecy in polyploid populations is derived from ancestral dioecy, and a study using experimental evolution has demonstrated the role that leakiness in sex expression has probably played in this transition (Cossard *et al.*, 2021). Clearly, hermaphroditism could only ever evolve from dioecy if males or females occasionally expressed both sex functions, either as a result of recombination between sterility loci on a young sex chromosome, thereby regenerating the ancestral hermaphrodite phenotype (Dorken and Barrett, 2004; Spigler *et al.*, 2008; Charlesworth, 2019; Massonnet *et al.*, 2020), or through leaky sex expression. The expression of leakiness as a result of simulated herbivory would thus represent a potentially interesting case of genetic assimilation, whereby a phenotypically plastic response first exposes a potentially advantageous phenotype to selection (Waddington, 1953). If the propensity to respond plastically varies genetically among individuals, as appears to be the case for leakiness in sex expression in *M. annua* (Cossard and Pannell, 2019), leakiness induced by simulated herbivory (or mate limitation: Cossard and Pannell, 2021) might then quickly become assimilated as established hermaphroditism in response to ongoing natural selection. Further work will be necessary to understand the details of this potential conversion from a plastic to an assimilated state.

## SUPPLEMENTARY DATA

Supplementary data are available online at https://academic.oup.com/aob and consist of Table S1: estimates based on model predictions.

mcab129_suppl_Supplementary_Table
