# Supplementary material for: Simulated herbivory enhances leaky sex expression in the dioecious herb Mercurialis annua
Source: Ann Bot. 2021 Oct 20;129(1):79–86. doi: 10.1093/aob/mcab129 (PMC8829902; doi:10.1093/aob/mcab129)
Supplement: mcab129_suppl_Supplementary_Table [file mcab129_suppl_Supplementary_Table.docx]

**SUPPLEMENTARY MATERIAL**

**Table S1.** Estimates based on model predictions: indicate are the mean, standard error and the lower and upper 95% confidence intervals.

| **Response variable** | **Sex** | **Treatment** | **Mean** | **SE** | **Lwr 95 CI** | **Upr 95 CI** |
| --- | --- | --- | --- | --- | --- | --- |
| **Leakiness probability** | Males | Low herbivory | 0.045 | 0.25 | 0.028 | 0.072 |
|  |  | High herbivory | 0.193 | 0.134 | 0.155 | 0.237 |
|  | Females | Control | 0.351 | 0.216 | 0.261 | 0.453 |
|  |  | Herbivory | 0.614 | 0.207 | 0.514 | 0.705 |
| **Number of seeds** | Males | Low herbivory | 0.241 | 0.562 | 0.080 | 0.723 |
|  |  | High herbivory | 1.806 | 0.312 | 0.981 | 3.325 |
| **Number of male flowers** | Females | Control | 0.271 | 0.324 | 0.143 | 0.513 |
|  |  | Herbivory | 1.366 | 0.262 | 0.816 | 2.287 |
